# Epratuzumab targeting of CD22 affects adhesion molecule expression and migration of B-cells in systemic lupus erythematosus

**DOI:** 10.1186/ar3179

**Published:** 2010-11-04

**Authors:** Capucine Daridon, Daniela Blassfeld, Karin Reiter, Henrik E Mei, Claudia Giesecke, David M Goldenberg, Arne Hansen, Arwed Hostmann, Daniela Frölich, Thomas Dörner

**Affiliations:** 1 Charite - Universitätsmedizin Berlin, CC12 Dept. Medicine/Rheumatology and Clinical Immunology, Chariteplatz 1, Berlin 10117, Germany; 2 Deutsches Rheumaforschungszentrum (DRFZ), Chariteplatz 1, Berlin 10117, Germany; 3 Center for Molecular Medicine and Immunology, Garden State Cancer Center, 520 Belleville Ave., Belleville, NJ 07109, USA

## Abstract

**Introduction:**

Epratuzumab, a humanized anti-CD22 monoclonal antibody, is under investigation as a therapeutic antibody in non-Hodgkin's lymphoma and systemic lupus erythematosus (SLE), but its mechanism of action on B-cells remains elusive. Treatment of SLE patients with epratuzumab leads to a reduction of circulating CD27^negative ^B-cells, although epratuzumab is weakly cytotoxic to B-cells *in vitro*. Therefore, potential effects of epratuzumab on adhesion molecule expression and the migration of B-cells have been evaluated.

**Methods:**

Epratuzumab binding specificity and the surface expression of adhesion molecules (CD62L, β7 integrin and β1 integrin) after culture with epratuzumab was studied on B-cell subsets of SLE patients by flow cytometry. In addition, *in vitro *transwell migration assays were performed to analyze the effects of epratuzumab on migration towards different chemokines such as CXCL12, CXCL13 or to CXCR3 ligands, and to assess the functional consequences of altered adhesion molecule expression.

**Results:**

Epratuzumab binding was considerably higher on B-cells relative to other cell types assessed. No binding of epratuzumab was observed on T-cells, while weak non-specific binding of epratuzumab on monocytes was noted. On B-cells, binding of epratuzumab was particularly enhanced on CD27^negative ^B-cells compared to CD27^positive ^B-cells, primarily related to a higher expression of CD22 on CD27^negative ^B-cells. Moreover, epratuzumab binding led to a decrease in the cell surface expression of CD62L and β7 integrin, while the expression of β1 integrin was enhanced. The effects on the pattern of adhesion molecule expression observed with epratuzumab were principally confined to a fraction of the CD27^negative ^B-cell subpopulation and were associated with enhanced spontaneous migration of B-cells. Furthermore, epratuzumab also enhanced the migration of CD27^negative ^B-cells towards the chemokine CXCL12.

**Conclusions:**

The current data suggest that epratuzumab has effects on the expression of the adhesion molecules CD62L, β7 integrin and β1 integrin as well as on migration towards CXCL12, primarily of CD27^negative ^B-cells. Therefore, induced changes in migration appear to be part of the mechanism of action of epratuzumab and are consistent with the observation that CD27^negative ^B-cells were found to be preferentially reduced in the peripheral blood under treatment.

## Introduction

Systemic lupus erythematosus (SLE) is a very heterogeneous autoimmune disease with various clinical manifestations and different immune abnormalities, including the production of a plethora of autoantibodies, deposition of immune complexes in various organs, and potential organ failure [1]. In patients with SLE, disturbances of B-cells in the peripheral blood (including an increase of CD27^negative ^transitional B-cells and CD27^positive ^B-cells as well as enhanced CD27^high ^plasmablasts), abnormalities of humoral immunity, immune complex formation, complement activation as well as experiences in clinical trials with B-cell directed therapy, suggest a key role for B-cells in the pathogenesis of this disease. Hence, immunotherapy targeting B-cells is currently of great interest with the promise to improve current treatments of SLE. In this context, epratuzumab, a humanized monoclonal IgG_1 _antibody (mAb) that targets the B-cell surface molecule CD22, has been explored in an early clinical trial [2] and more recently in a phase IIb randomized clinical study which showed a treatment advantage with epratuzumab over placebo of around 25% at week 12 [3].

CD22, a 140 kDa transmenbrane type 1 protein, also called Sialic acid-binding Ig-like lectin 2 (Siglec-2) or B-lymphocyte cell adhesion molecule (BL-CAM), is a member of the Siglec family that binds to α2-6-linked sialic acids on glycoproteins. These ligands for CD22 are widely expressed on different cell types [4] (co called *trans *glycoprotein ligands) including B-cells (where CD22 will also bind *cis *glycoprotein ligands).

CD22 is differentially expressed during B-cell differentiation. At early developmental stages, such as pre-B-cells, CD22 is expressed intracellularly and appears on the surface on immature B-cells reaching the highest surface expression levels on mature B-cells and declining substantially during final maturation into plasma cells [5-7]. Although Stathish *et al. *also described the expression of CD22 on murine primary T-cells [8], CD22 has not been detected on human T-cells and monocytes [4].

Interestingly, CD22 has two different functions on B-cells. It is well known as a negative regulatory molecule of the B-cell antigen receptor (BCR) signal leading to inhibition of B-cell activation by phosphorylation of the protein tyrosine phosphatase SHP-1 (Src homology region 2 domain-containing phosphatase 1) via the immunoreceptor tyrosine-based inhibitory motifs (ITIMs) contained in the cytoplasmic tail [9]. Moreover, CD22 is also considered as an adhesion receptor for the homing of re-circulating IgD^positive ^B-cells in the bone marrow via the expression of CD22 ligand on bone marrow sinusoidal endothelium [10-12].

The functional diversity of CD22 has implications for the hitherto unknown mechanism of action by epratuzumab and is of interest. Initial treatment with this mAb in patients with SLE showed a significant decrease of BILAG (British Isles Lupus Assessment Group) scores above 50% [2]. In this study, a significant reduction of peripheral B-cells was also observed in SLE patients who were treated with epratuzumab, primarily a 30% reduction of CD27^negative ^B-cells comprising transitional and naïve B-cells [2,13]. The reason for the reduction in B-cell numbers remains unknown.

In this context, earlier studies reported that epratuzumab, in contrast to rituximab, was weakly cytotoxic for B-cells since it could induce modest antibody-dependent cellular cytotoxicity (ADCC) and no complement-dependent cytotoxicity (CDC) *in vitro*; however, epratuzumab modulates exaggerated activation and proliferation of B-cells from SLE patients following CpG, BCR and CD40L stimulation [13-15]. Epratuzumab binds to non-ligand binding epitopes on CD22 and provokes phosphorylation of CD22 [16,17].

While epratuzumab appears to have only a very limited capacity to induce direct apoptosis [13,14] via CDC and ADCC, the apparent reduction of peripheral blood CD27^negative ^B-cells under therapy led to the hypothesis that triggering CD22 could modulate B-cell migration possibly resulting in reduced CD27^negative ^B-cell counts in the blood. Since cell trafficking is a multistep process involving the concerted interaction of cell adhesion molecules binding to their respective ligands as well as chemokine-regulated migration pathways, our study was designed to assess the effects of epratuzumab on the expression of a range of adhesion molecules (CD62L, β1 integrin and β7 integrin) and migration towards CXCL12, CXCL13 and a number of CXCR3 ligands (CXCL9, 10 and 11) on peripheral blood mononuclear cells (PBMCs) from SLE patients. These three adhesion molecules and their ligands are critical for B-cell trafficking. CD62L (L-selectin) is involved in the homing of B-cells preferentially into peripheral lymphoid tissues through high endothelial venules (HEV). The β7 integrin associated with its α4 integrin partner (to form the α4β7 integrin) is responsible for the homing of lymphocytes preferentially into mucosal immune tissues via the ligand mucosal addressin cell adhesion molecule-1 (MAdCAM-1) on large endothelial venules, while the α4β1 integrin, a receptor for fibronectin and vascular cell adhesion molecule-1 (VCAM-1), is preferentially involved in the homing and retention of lymphocytes and hematopoietic stem cells to the bone marrow and the trafficking of leukocytes [18-23]. Therefore, we addressed the potential influence of epratuzumab on the cell surface expression of adhesion molecules and cell migration *in vitro *which are important B-cell functions.

The results from the current study demonstrate specific binding of epratuzumab on B-cells. Additionally, we observed greater binding of epratuzumab on CD27^negative ^B-cells which was related to the expression of CD22 which was higher on CD27^negative ^B-cells compared to CD27^positive ^B-cells. Epratuzumab binding to CD27^negative ^B-cells induced a significant reduction of CD62L and β7 integrin surface expression, while β1 integrin was up-regulated. Functionally, CD27^negative ^B-cells cultured with epratuzumab showed enhanced spontaneous migration across fibronectin coated filters. Finally, epratuzumab incubation was found to enhance the migration towards CXCL12 of CD27^negative ^B-cells, but not of CD27^positive ^B-cells. These results suggest that epratuzumab is able to modulate B-cell migration and adhesion molecule expression, processes that potentially contribute to its mechanism of action in SLE.

## Materials and methods

### Subjects

After informed consent was obtained for the protocol approved by the Institutional Review Board at the Charité - University Hospitals, Berlin, SLE patients were enrolled in the study. All patients fulfilled the American College of Rheumatology ACR criteria, revised in 1982 [24]. Thirty SLE patients (28 females, 2 males), 39.1 ± 13.9 years old were studied. PBMCs were prepared from 30 to 40 mL anti-coagulated blood by density gradient centrifugation over ficoll-paque (Amersham Pharmacia Biotech, Uppsala, Sweden), and then washed twice with phosphate-buffered saline (PBS) supplemented with 0.05% (w/v) of bovine serum albumin (BSA, Sigma-Aldrich, Seelze, Germany).

### Adhesion molecule surface expression after epratuzumab incubation

To monitor changes of adhesion molecule surface expression (β1 integrin, β7 integrin and CD62L) after epratuzumab incubation, freshly isolated PBMCs were incubated with 10 μg/mL of epratuzumab in RPMI 1640 medium (Gibco BRL, Karlsruhe, Germany) supplemented with 0.5% (w/v) BSA for 90 minutes at 37°C and 5% CO_2_. After incubation, the PBMCs were washed in cold PBS-BSA 0.05% (w/v) and then stained on ice for FACS analysis as described below.

### Fibronectin-dependent chemotaxis

Fibronectin-dependent chemotaxis was assessed using transwell migration plates (5.0 μm pores, Corning Life Sciences, Acton, MA, USA) coated with 10 μg/mL of fibronectin (Invitrogen, Carlsbad, CA, USA), a ligand for the β1 integrin [25].

1 × 10^6 ^PBMCs were incubated with or without 10 μg/mL of epratuzumab and allowed to migrate for 90 minutes at 37°C and 5% CO_2 _using transwell migration assays. Migration towards CXCL12 (50 nM) (stromal cell-derived factor, SDF1) or CXCL13 (250 nM) (B-cell homing chemokine, BLC or also B-cell attracting chemokine 1, BCA1) or to a mix of CXCR3 ligands (CXCL9 (250 nM) (monokine induced by gamma interferon, MIG), CXCL10 (300 nM) (interferon inducible protein 10, IP10) and CXCL11 (10 nM) (interferon-inducible T-cell alpha chemoattractant, I-TAC)) were studied by adding the different chemokines to the lower chamber in RPMI 1640 supplemented with 0.5% (w/v) BSA as described previously [26]. All chemokines were from R&D Systems, Minneapolis, MN, USA.

At the end of the incubation, migrated and non-migrated cells were harvested from the lower and upper compartments, respectively, counted and phenotyped by FACS as described below. The results were expressed as percentage of migrated B-cells using the following formula: number of migrated B-cells/(number of non migrated B-cells + number of migrated B-cells) × 100.

To assess spontaneous migration, controlled migrations were performed without using any chemokine gradient. The B-cells that migrated independently of the chemokine gradient were considered to have functional β1 integrin.

### FACS analysis

Staining of freshly isolated PBMCs and treated PBMCs was performed as described previously [26]. The following antibodies were used: CD3-Pacific Blue (PB) or H7-allophycocyanin (APC) (BD, Clone UCHT1), CD14-PB or H7-APC (BD, Clone m5e2), CD19-phycoerythrin-cyanin 7 (PE-Cy7) (BD, Clone SJ 25C1), CD20-peridin chlorophyll protein (PerCP) (BD, Clone L27), CD62L-fluorescein isothiocyanate (FITC) (Clone 145/15, Miltenyi Biotec, Auburn, CA, USA), CD27-cyanin 5 (Cy5) (clone 2E4, kindly provided by Rene Van Lier, University of Amsterdam, The Netherlands), β7 integrin-phycoerythrin (PE) (BD, clone FIB504), β1 integrin-PE (BD, clone MAR4), CD22-PE (BD, clone S-HCL-1) and epratuzumab IgG and F(ab')_2 _fragment of epratuzumab (provided by UCB, Slough, UK). T-cells, B-cells and monocytes were gated using their scatter properties and stained for CD3, CD19 or CD14. Analysis was performed with a Becton Dickinson Canto II machine and data were analyzed using FCS Express 3.0 software (DeNovo Software, Los Angeles, CA, USA) or using FlowJo™ software (TreeStar, Ashland, OR, USA).

### Binding specificity of epratuzumab experiments

A total of 1 to 2 × 10^6 ^freshly isolated PBMCs were pre-incubated in 50 μl of PBS/0.05% (w/v) BSA with or without 8.8 μg of unlabeled F(ab')_2 _fragment of epratuzumab for 10 minutes on ice, then 1 μg of PE-labeled epratuzumab, CD3/14 H7-APC, CD27-Cy5, CD20-PerCP and CD19-PE-Cy7 were added to the PBMCs. After 15 minutes of staining in the dark, the PBMCs were washed two times in cold PBS/0.05% (w/v) BSA and then analyzed by FACS.

### Statistical analysis

Unpaired data sets were compared using the nonparametric Mann-Whitney U-test and paired data were analyzed using the Wilcoxon test with GraphPad Prism4 software (GraphPad, San Diego, CA, USA). A *P-*value less than 0.05 was considered significant (* *P *< 0.05; **P < 0.01; ***P < 0.001). All values are expressed as mean ± standard deviation unless otherwise specified.

## Results

### Enhanced CD22 expression and epratuzumab binding to CD27^negative ^B-cells from SLE patients

**Figure 1:**
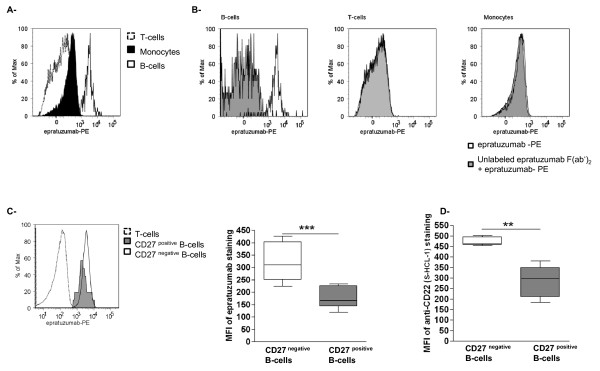
**The binding capacity of epratuzumab on different PBMCs obtained from SLE patients**. **(a) **FACS analyses were performed on PBMCs from SLE patients using PE- labeled epratuzumab. Representative histogram of the differential binding of epratuzumab on T-cells (CD3^positive^, dotted line), monocytes (CD14^positive^, black histogram) and B-cells (CD19^positive^, black line). **(b) **PBMCs were incubated with (grey histogram) or without (black line) unlabelled F(ab')_2 _epratuzumab fragment for 10 minutes at 4°C. PBMCs were then stained with PE labeled-epratuzumab, and epratuzumab binding analyzed on B-cells, T-cells and monocytes (*n *= 3). Representative histogram of epratuzumab binding on B-cell sub-populations: CD27^negative ^B-cells (black line), CD27^positive ^B-cells (grey histogram) and T-cells (negative control, dotted line) are shown in **(c)**. The results of the FACS analysis (right graph), showed higher binding capacity of epratuzumab on CD27^negative ^B-cells compare to CD27^positive ^B-cells (*P *= 0.0002). **(d) **To study the expression of CD22 on B-cells, PBMCs were stained with a mouse anti-CD22 mAb (Clone S-HCL-1), which recognizes a different epitope than epratuzumab (*n *= 5) [16]. The FACS analysis demonstrated that CD22 is more highly expressed on CD27^negative ^B-cells compared to CD27^positive ^B-cells.

In order to delineate more thoroughly the effects of epratuzumab in relation to its target CD22, the binding capacity of epratuzumab to specific leukocyte subsets such as T-cells, B-cells and monocytes was studied. Therefore, FACS analyses were performed on PBMCs from SLE patients with PE-labelled epratuzumab. Clear binding of epratuzumab on B-cells was shown, whereas no epratuzumab binding was observed on T-cells. Interestingly, epratuzumab appeared to bind to monocytes (Figure 1a). To further evaluate the binding specificities of epratuzumab, we performed blocking experiments where cells were incubated with unlabeled F(ab')_2 _fragments of epratuzumab for 10 minutes on ice (Figure 1b, grey histogram) and then stained with PE-labeled epratuzumab (Figure 1b, black line histogram). We observed inhibition of epratuzumab binding after incubation with unlabeled F(ab')_2 _fragments of epratuzumab on B-cells (Figure 1b, left graph). From these experiments, we conclude that epratuzumab binds specifically to B-cells via CD22 without a requirement for Fc fragment binding. T-cells did not show any epratuzumab binding and were subsequently used as negative control. Notably, we did not observe any significant inhibition of epratuzumab binding on monocytes after incubation with unlabeled F(ab')_2 _fragment of epratuzumab (Figure 1b), suggesting that the binding of this antibody to monocytes is likely related to the Fc moiety. Furthermore, experiments with a commercially available mouse anti-human CD22 antibody, clone S-HCL-1, targeting a different epitope on CD22 than epratuzumab [16] did not show any binding to either monocytes or T cells (data not shown). These results confirmed the absence of surface expression of CD22 on T-cells and monocytes as described by others [4].

Subsequent studies focused in detail on the effects induced by epratuzumab on particular B-cell subpopulations. Initially, we studied the expression of CD22 on B cell subsets based on their expression of CD27, the CD27^negative ^B-cell subpopulation comprising naïve and transitional B-cells and the CD27^positive ^B-cells comprising pre- (IgD^+^) and post-switch (IgD^-^) memory B-cells [13].

In this analysis, a substantially higher binding of epratuzumab on CD27^negative ^B-cells versus CD27^positive ^B-cells was observed as shown by a representative histogram in Figure 1c. In fact, there was a two-fold enhanced anti-CD22 binding to CD27^negative ^B-cells (mean fluorescence intensity (MFI) 324.7 ± 74.4) compared to CD27^positive ^B-cells (MFI 172.9 ± 40.9) (*P *= 0.0002).

In order to confirm the differential expression of CD22 on B-cell subpopulations, experiments were repeated using the mouse anti-human CD22 antibody, S-HCL-1, which recognizes a different epitope to epratuzumab [16]. These experiments confirmed a higher CD22 expression on CD27^negative ^B-cells compared to CD27^positive ^B-cells (Figure 1d, *n *= 5). In summary, specific binding of epratuzumab on the surface of B-cells has been confirmed with the highest propensity identified on CD27^negative ^B-cells.

### Epratuzumab down-modulates CD62L and β7 integrin surface expression on B-cells

Subsequent studies were designed to identify whether epratuzumab binding to CD22, known to function as an adhesion molecule, could modulate the surface expression of other adhesion molecules on B-cells. Therefore, CD62L, β7 integrin and β1 integrin surface expression on PBMCs from SLE patients were studied *in vitro *after culture with epratuzumab.

**Figure 2:**
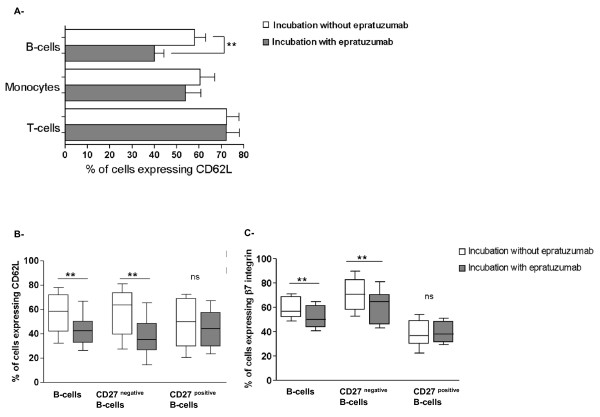
**Epratuzumab leads to decreased surface expression of the adhesion molecules CD62L and β7 integrin on CD27^negative ^B-cells**. Comparison of the surface expression of CD62L on PBMCs from SLE patients with (grey histogram) and without (white histogram) epratuzumab incubation **(a)**. Monocytes (CD14^positive^) showed a moderate but non-significant reduction of CD62L, whereas this expression was not influenced on T-cells (CD3^positive^) by epratuzumab. Notably, epratuzumab led to a significant reduction of the CD62L surface expression on B-cells (*P <*0.01). These comparative studies of CD27^negative ^B-cells versus CD27^positive ^B-cells demonstrated that the reduction of CD62L was confined to CD27^negative ^B-cells **(b) **(*P <*0.01). Similarly, the surface expression of β7 integrin **(c) **was significantly reduced by epratuzumab on CD27^negative ^B-cells but not on CD27^positive ^B-cells (** *P *< 0.01; ns not significant, *n *= 9).

First, the surface expression of CD62L, an adhesion molecule involved in systemic B-cell activation [19], on PBMCs was assessed. As shown in Figure 2a (representative of nine independent experiments using PBMCs from SLE patients), epratuzumab incubation led to a significant down-modulation of CD62L on the surface of B-cells (*P *= 0.0078). Thus, CD62L was found to be expressed on 56.7 ± 16.4% of peripheral B-cells after incubation without epratuzumab which was reduced to 42.5 ± 12.6% after epratuzumab incubation. Notably, around 15% of B-cells became negative for CD62L expression on their surface after epratuzumab incubation. No significant difference was observed either on peripheral T-cells or monocytes after epratuzumab incubation (Figure 2a).

Further studies demonstrated that the reduced surface expression of CD62L was restricted to CD27^negative ^B-cells; when this subset was analyzed, 57.9 ± 18.6% were positive for CD62L in the absence of epratuzumab (white bar, Figure 2b) and 37.9 ± 15.5% after epratuzumab incubation (grey bar, Figure 2b) (**P = 0.004; Figure 2b). However, the frequency of B-cells being CD62L^positive ^and CD27^positive ^remained unaffected by epratuzumab (48.7 ± 19% *versus *44 ± 15.4%).

Additional studies on the expression of the mucosal adhesion molecule β7 integrin on CD27^positive ^and CD27^negative ^B-cells are summarized in Figure 2c. Epratuzumab incubation induced a significant reduction of the surface expression of β7 integrin (*P *= 0.0039), primarily confined to CD27^negative ^B-cells, while no changes were observed on CD27^positive ^B-cells. Of note, surface expression of CD62L and β7 integrin was simultaneously down-modulated on B-cells after incubation with epratuzumab. The percentage of CD27^negative ^B-cells expressing both CD62L and β7 integrin was significantly decreased after epratuzumab incubation, from 44.5 ± 16.6% to 28.1 ± 15% (data not shown). The decrease of both CD62L and β7 integrin on the surface of CD27^negative ^B-cells suggests that epratuzumab has the potential to change the adhesion characteristics of this particular population.

### Epratuzumab incubation leads to an increase of β1 integrin surface expression on B-cells

**Figure 3:**
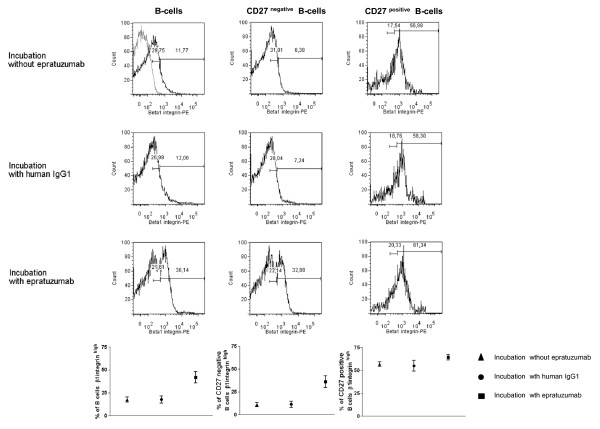
**Epratuzumab induces β1 integrin on CD27^negative ^B-cells**. In order to analyse changes in the surface expression of β1 integrin on B-cell sub-populations (CD27^negative ^B-cells and CD27^positive ^B-cells), we incubated PBMCs for 90 minutes at 37°C, 5% CO_2 _without epratuzumab (first line), or with human IgG_1 _(second line) or with epratuzumab (third line) (*n *= 3). The histograms are representative of three independent experiments and the results are summarized in Figure 3 (mean ± SEM).

Based on the observed down-modulation of β7 integrin surface expression on CD27^negative ^B-cells by epratuzumab, further analyses focused on the effect of epratuzumab on the expression of the β1 integrin on the surface of B-cells. In this regard, integrin complexes are composed of β and α subunits and form unique molecules, and β7 integrin essentially competes with β1 integrin for the same α4 subunit. A recent study [27] reported that the surface expression of α4β7 integrin is regulated in a homeostatic relation with the surface expression of α4β1 integrin on T-cells since high expression of α4β1 integrin resulted in a loss of α4β7 integrin. With this in mind, subsequent studies were performed to analyze whether down-modulation of β7 integrin on the surface of the CD27^negative ^B-cells is associated with changes in the expression of β1 integrin. There appeared to be two populations of B-cells that, in their basal state, were either β1 integrin^low ^or β1 integrin^high ^as shown in Figure 3. In fact, the data showed that the majority of CD27^positive ^B-cells expressed high basal levels of β1 integrin on their cell surface compared with CD27^negative ^B-cells (Figure 3, middle and right column). After incubation of PBMCs with epratuzumab, the percentage of B-cells that were β1 integrin^high ^increased from 17 ± 7% to 42 ± 10.5% (Figure 3). With regard to CD27^negative ^B-cells, a significant change in the proportion of β1 integrin^high ^cells was observed after incubation with epratuzumab (36.3 ± 11.3%) versus a human IgG_1 _control (11.4 ± 6.3%) or without Ig (11.2 ± 3.9%). No difference in the proportion of β1 integrin^high ^cells was observed on CD27^positive ^B-cells after epratuzumab treatment.

**Figure 4:**
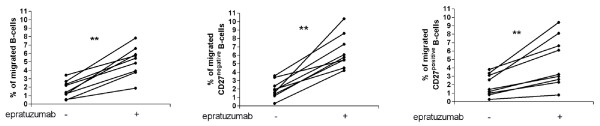
**Epratuzumab increases the motility of the B-cells**. To evaluate the functionality of β1 integrin on the B-cells, we checked the capacity of B-cells to transmigrate through fibronectin independently of chemoattractant, with epratuzumab incubation (data from nine independent experiments are shown in Figure 4). Epratuzumab caused an enhanced transmigration through fibronection layers; indeed, a threefold greater basal motility of treated CD27^negative ^B-cells was observed.

Since fibronectin is one of the ligands for the α4β1 integrin, transwell migration assays using fibronectin-coated filters were employed as functional tests for spontaneous migration. Notably, incubation of B-cells with epratuzumab provoked enhanced spontaneous transmigration of B-cells (Figure 4) which was seen for CD27^positive ^and CD27^negative ^B-cells. However, the transmigration was around threefold higher for CD27^negative ^B-cells compared to a twofold increase for CD27^positive ^B-cells.

These data suggest that epratuzumab is able to increase β1 integrin expression on a fraction of CD27^negative ^B-cells and this is associated with a substantial increase in the functional activity of this integrin.

### Epratuzumab enhanced migration of B-cells towards CXCL12

**Figure 5:**
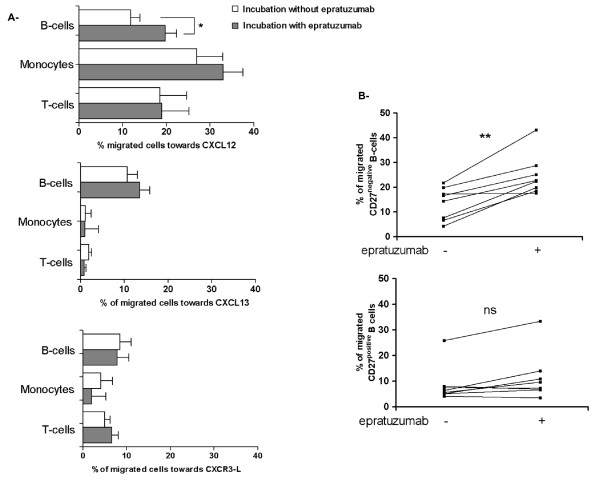
**Enhanced migration of CD27^negative ^B-cells from SLE patients towards CXCL12 after epratuzumab incubation**. To assess the migration towards CXCL12, CXCL13 and CXCR3 ligands of PBMCs from eight SLE patients, we performed transwell migration assays after epratuzumab incubation (10 μg/mL for 90 minutes). The migration of different cell types towards CXCL12 was analyzed by flow cytometry [(T-cells, CD3^positive^), (monocytes, CD14^positive^), (total B-cells CD19^positive^); CD27^negative ^B-cells CD27^positve ^B-cells] and epratuzumab incubation lead to a significantly enhanced migration of B-cells **(a) **(*P < 0.05). No effect of epratuzumab was observed on T-cells and on monocytes. Migration of all cell types, including B-cells, towards CXCL13 and CXCR3 ligands was unaffected by epratuzumab. **(b) **Studies of CD27^negative ^and CD27^positive ^B-cells from SLE patients revealed that the increased migration toward CXCL12 was primarily restricted to CD27^negative ^B-cells (** *P *< 0.01).

Additional experiments analyzed the effect of epratuzumab on the migration of B cells towards a range of chemokines, such as CXCL12, CXCL13 and to CXCR3 ligands. PBMCs from SLE patients were incubated with or without epratuzumab and allowed to migrate for 90 minutes at 37°C. Notably, epratuzumab further increased the chemotactic response towards CXCL12 (Figure 5a) (*P *= 0.015) but there was no significant change in the migration towards CXCL13 or CXCR3 ligands (Figure 5a). No influence of epratuzumab was noted on monocytes or T-cells. Furthermore, epratuzumab led to a more pronounced effect on the migration towards CXCL12 on CD27^negative ^B-cells compared to CD27^positive ^B-cells (*P *= 0.0078; Figure 5b). The migration capacity of both CD27^negative ^B-cells and CD27^positive ^B-cells to CXCL13 and CXCR3 ligands was unaffected by incubation with epratuzumab (data not shown). These data indicate that another consequence of high CD22 expression on CD27^negative ^B-cells is increased migration towards CXCL12 in the presence of epratuzumab.

## Discussion

This study demonstrates that epratuzumab is able to induce significant functional changes on B-cells *in vitro*, particularly of CD27^negative ^B-cells, which include 1) a substantial reduction of the cell-surface expression of CD62L and β7 integrin; 2) an associated increased β1 integrin cell-surface expression; and 3) enhanced spontaneous migration and directed migration towards CXCL12. Since such changes were not induced to a large extent by epratuzumab on CD27^positive ^B-cells, it appears plausible that these effects are linked to the higher CD22 surface expression on CD27^negative ^B-cells.

### Differential binding of epratuzumab to PBMCs

Epratuzumab was found to bind to the highest extent on CD27^negative ^B-cells followed by CD27^positive ^cells. The competition experiments demonstrated that this binding is specific via targeting CD22 and a role for Fc receptor binding could not be demonstrated. This difference between CD27^negative ^B-cells and CD27^positive ^B-cells can be explained by the higher expression of CD22 which we observed by FACS analysis and, according to the data base, CD22 mRNA is also more highly expressed on CD27^negative ^naïve B-cells and CD27^negative ^transitional B-cells than on CD27^positive ^B-cells (Platform ID: GPL570, accession no. [GEO:GSE17186]; Human B-cell subsets).

CD22 is not expressed on monocytes [4,6] but we did detect a small degree of epratuzumab binding to these cells consistent with the capacity of monocytes to mediate Fc receptor-dependent binding to antibodies [28,29].

### Modification of surface adhesion molecule expression by epratuzumab

We observed a number of changes in adhesion molecule expression on the surface of B-cells under the influence of epratuzumab, such as a decrease of CD62L on CD27^negative ^B-cells. Although the biological consequences of reduced CD62L expression remain to be delineated, it could potentially result in disturbed immune activation. Previous studies in CD62L-deficient mice reported that lymphocyte recruitment into inflammatory sites was inhibited significantly, whereas lymphocyte recruitment to the spleen was increased [30,31]. These results support the notion that reduced CD62L expression on B-cells by epratuzumab may disturb recruitment of B-cells to different sites of inflammation.

As with CD62L expression, epratuzumab incubation down-modulated β7 integrin surface expression which was again seen primarily on CD27^negative ^B-cells. The interaction of α4β7 integrin with its natural ligand, MAdCAM-1, is involved in the migration of immune cells to mucosal tissues. Whether there is a role for mucosal immune activation in SLE remains a matter of debate, although enhanced soluble CD14 likely related to LPS-dependent activation in gut-associated lymphoid tissues has been identified in the blood of SLE patients [32], consistent with persistent, enhanced activation of mucosal immunity.

Because of the apparent striking reductions of β7 integrin expression on CD27^negative ^B-cells after incubation with epratuzumab, and the known interdependence between β1 and β7 integrin expression on human T-cells which share the α4 integrin subunit [27], the regulation of β1 integrin expression after epratuzumab incubation was investigated. Of importance, epratuzumab induced β1 integrin expression on CD27^negative ^B-cells while its expression on the surface of CD27^positive ^B-cells was already high at baseline and not substantially changed by epratuzumab. The data indicate that β1 integrin and β7 integrin expression could be regulated interdependently on CD27^negative ^B-cells. In addition, binding to CD22 by epratuzumab provoked the activation of β1 integrin and resulted in enhanced spontaneous migration of both CD27^negative ^and CD27^positive ^B-cells over fibronectin-coated membranes, independent of any chemokine. Thus, epratuzumab-enhanced β1 integrin expression leads to functionally relevant effects on B-cells.

Overall, the modification of the adhesion molecule profile affected preferentially a fraction of CD27^negative ^B-cells. In this context, CD27^negative ^B-cells comprise two main B-cell sub-populations, transitional B-cells and mature naïve B-cells [33]. However, we were unable to distinguish between these two subpopulations in our experiments. Since expansion of transitional B-cells in the circulation is correlated with clinical responses assessed by SLEDAI (Systemic Lupus Erythematosus Disease Activity Index) [33], further experiments are warranted to evaluate this hypothesis.

### Enhanced migration of CD27^negative ^B-cells to CXCL12 in the presence of epratuzumab

Studies conducted to investigate changes on B-cell migration showed that epratuzumab is able to enhance the migration of CD27^negative ^B-cells across gradients of CXCL12 *in vitro*. This chemokine has been shown to be expressed by lymphoid organs [34,35] and inflamed kidneys [36,37] and, therefore, may account for enhanced emigration of CD27^negative ^B-cells but not memory CD27^positive ^B-cells from the peripheral blood observed clinically in SLE patients treated with epratuzumab [13]. Since epratuzumab binds preferentially to CD27^negative ^B-cells, one could speculate that the enhanced migration of CD27^negative ^B-cells is due to a stronger effect of CD22 expression on these cells.

Moreover, the bone marrow produces substantial amounts of CXCL12, and is able to attract antibody-secreting cells from the periphery [38]; it also employs CD22 and β1 integrin for migration and retention of B-cells to this site [6,11,12,21,23]. If similar mechanisms apply also for other B cell subpopulations, such as CD27^negative ^transitional or CD27^negative ^naïve B-cells, it is also possible that these cells become trapped in the bone marrow and cannot fully differentiate in secondary lymphoid organs.

Epratuzumab did not influence B-cell migration towards CXCL13 and CXCR3 ligands using PBMCs from SLE patients which argues that the migration changes observed with epratuzumab are not non-specific. However, we cannot rule out the possibility that the enhanced migration of CD27^negative ^B-cells obtained from SLE patients may reflect the loss of migrating cells from the peripheral blood during active lupus. In this regard, it has been reported that levels of CXCL9, CXCL10 and CXCL11 correlate with lupus disease activity [39,40]. In addition, CD4^positive^CXCR3^positive ^T-cells have been found to be increased in the urine of patients with lupus nephritis, which may suggest involvement of this chemokine system [37].

With regard to the current study, a discussion of the interrelationship between CD22 modulation by epratuzumab and changes in adhesion molecule expression and migration is of importance. Interactions between intracellular signaling pathways may offer an explanation. In this context, Lyn is known to be the kinase responsible for phosphorylation of ITIMs on CD22 [41] and it has been reported that Lyn is of critical importance in SLE with lower expression of Lyn being typical for SLE patients [42,43]. In addition, Lyn is closely related to Syk (Spleen tyrosine kinase) which is involved in BCR signaling, but also in the regulation of integrin activation [44]. Moreover, recent work indicates that Syk is involved in CXCL12- and α4β1 integrin-induced signaling in chronic lymphocytic leukemia [45]. Further investigation of CD22-Lyn-Syk interactions could lead to a better understanding of the precise mechanisms by which CD22 regulates cell adhesion and migration pathways in B cells.

## Conclusions

This study demonstrates for the first time that the humanized anti-CD22 mAb, epratuzumab, displays a substantially higher degree of binding to CD22 on CD27^negative ^B-cells resulting in functional effects such as enhanced migration towards CXCL12 and modification of adhesion molecule cell surface expression (down-regulation of the expression of CD62L and β7 integrin and up-regulation of β1 integrin). Collectively, the data suggest that epratuzumab could disturb trafficking of CD27^negative ^B-cells, which provides an insight into the potential mechanism of action of this antibody in SLE.

## Abbreviations

ACR: American College of Rheumatology; ADCC: antibody-dependent cellular cytotoxicity; ASC: antibody-secreting cells; BCR: B-cell antigen receptor; BILAG: British Isles Lupus Assessment Group; SLEDAI: Systemic Lupus Erythematosus Disease Activity Index; BL-CAM: B-lymphocyte cell adhesion molecule; BSA: bovine serum albumin; CDC: complement-dependent cytotoxicity; Cy5: cyanin 5; DAPI: 4',6-diamidino-2-phenylindol; F(ab')_2_: fragments antigen binding 2; FACS: fluorescence activated cell sorting; FITC: fluorescein isothiocyanate; H7-APC: H7-allophycocyanin; ITIMs: immunoreceptor tyrosine-based inhibitory motifs; mAb: monoclonal antibody; MFI: mean fluorescence intensity; MIG: monokine induced by gamma interferon; PB: pacific blue; PBMCs: peripheral blood mononuclear cells; PBS: phosphate-buffered saline; PE: phycoerythrin; PE-Cy7: phycoerythrin-cyanin 7; PerCP: peridin chlorophyll protein; Siglec-2: Sialic acid-binding Ig-like lectin 2; SLE: systemic lupus erythematosus; SyK: spleen tyrosine kinase; VCAM-1: vascular cell adhesion molecule-1.

## Competing interests

TD has received research support from Immunomedics. This study was funded in part by UCB Inc; DMG has a management role and equity in Immunomedics, Inc. All other authors declare that they do not have any competing interests.

## Authors' contributions

DB, CD, KR, DF, AHo carried out experimental work in different areas. AHa, CG and AHo discussed the data at several stages and worked on the manuscript. CD, DMG, HM, and TD designed the study, analyzed data and wrote the manuscript. All authors read and approved the final manuscript.
